# Supplementary material for: Peanut and Peanut-Based Foods Contamination by Toxigenic Fungi and Mycotoxins: Potential Risks for Beninese Consumers
Source: Toxins (Basel). 2025 Oct 29;17(11):532. doi: 10.3390/toxins17110532 (PMC12656416; doi:10.3390/toxins17110532)
Supplement: Supplementary file 1 [file toxins-17-00532-s001.zip › toxins-3931370-supplementary.pdf]

Supplementary Materials: Peanut and Peanut-Based Foods Contamination by Toxigenic Fungi and Mycotoxins: Potential Risks for Beninese Consumers

Christin Sogbossi Gbétokpanou, Camille Jonard, Ornella Anaïs Mehinto, Sébastien Gofflot, Mawougnon Jaurès Martial Adjéniya, Ogouyôm Herbert Iko Afe, Dona Gildas Anihouvi, Samiha Boutaleb, Claude Bragard, Paulin Azokpota, Jacques Mahillon, Marianne Sindic, Marie-Louise Scippo, Yann Eméric Madodé and Caroline Douny

Table S1: Mycotoxins concentrations of marketed roasted peanut snacks and *kluiklui* (µg/kg)

Category	Sample ID	AFB1	AFB2	AFG1	AFG2	AOH	FUM B1	FUM B2	FUM B3	OTA	OTB	STR
Roasted peanut snacks	20230.0S259	1	<LOD	<LOD	<LOD	<LOD	<LOD	<LOD	<LOD	< LOQ	< LOQ	< LOQ
Roasted peanut snacks	20230.0S260	< LOQ	<LOD	<LOD	<LOD	<LOD	<LOD	<LOD	<LOD	< LOQ	< LOQ	< LOQ
Roasted peanut snacks	20230.0S261	2	<LOD	<LOD	<LOD	<LOD	<LOD	<LOD	<LOD	< LOQ	< LOQ	< LOQ
Roasted peanut snacks	20230.0S263	7	<LOD	<LOD	<LOD	<LOD	<LOD	<LOD	<LOD	< LOQ	< LOQ	< LOQ
Roasted peanut snacks	20230.0S264	4	<LOD	<LOD	<LOD	<LOD	<LOD	<LOD	<LOD	5085	2143	< LOQ
Roasted peanut snacks	20230.0S265	< LOQ	<LOD	<LOD	<LOD	<LOD	<LOD	<LOD	<LOD	5	< LOQ	< LOQ
Roasted peanut snacks	20230.0S266	< LOQ	<LOD	<LOD	<LOD	<LOD	<LOD	<LOD	<LOD	< LOQ	< LOQ	< LOQ
Roasted peanut snacks	20230.0S269	169	<LOD	<LOD	<LOD	<LOD	<LOD	<LOD	<LOD	3	< LOQ	3
Roasted peanut snacks	20230.0S258	2.2	0.7	<LOD	<LOD	<LOD	<LOD	<LOD	<LOD	<LOD	<LOD	<LOD
Roasted peanut snacks	20230.0S262	<LOD	<LOD	<LOD	<LOD	<LOD	<LOD	<LOD	<LOD	<LOD	<LOD	<LOD
Roasted peanut snacks	20230.0S267	<LOD	<LOD	<LOD	<LOD	<LOD	<LOD	<LOD	<LOD	<LOD	<LOD	<LOD
Roasted peanut snacks	20230.0S268	<LOD	<LOD	<LOD	<LOD	<LOD	<LOD	<LOD	<LOD	<LOD	<LOD	<LOD
Roasted peanut snacks	20230.0S270	4.7	1.4	<LOD	<LOD	<LOD	<LOD	<LOD	<LOD	<LOD	<LOD	<LOD
Roasted peanut snacks	20230.0S271	0.5	< LOQ	0.4	<LOD	<LOD	<LOD	<LOD	<LOD	<LOD	<LOD	<LOD
Roasted peanut snacks	20230.0S272	1.9	0.6	<LOD	<LOD	<LOD	<LOD	<LOD	<LOD	<LOD	<LOD	<LOD

Roasted peanut snacks	20230.0S273	18	5.1	0.4	<LOD	<LOD	<LOD	<LOD	<LOD	<LOD	<LOD	<LOD
Roasted peanut snacks	20230.0S274	14	3.6	0.9	<LOD	<LOD	<LOD	<LOD	<LOD	<LOD	<LOD	<LOD
Roasted peanut snacks	20230.0S275	<LOD	<LOD	<LOD	<LOD	<LOD	<LOD	<LOD	<LOD	<LOD	<LOD	<LOD
Roasted peanut snacks	20230.0S276	0.3	<LOD	<LOD	<LOD	<LOD	<LOD	<LOD	<LOD	<LOD	<LOD	<LOD
Roasted peanut snacks	20230.0S277	14	4.2	1	0.5	<LOD	<LOD	<LOD	<LOD	<LOD	<LOD	<LOD
Roasted peanut snacks	20230.0S278	5.4	1.9	<LOD	<LOD	<LOD	<LOD	<LOD	<LOD	<LOD	<LOD	<LOD
Roasted peanut snacks	20230.0S279	< LOQ	<LOD	<LOD	<LOD	<LOD	<LOD	<LOD	<LOD	<LOD	<LOD	<LOD
Roasted peanut snacks	20230.0S280	0.46	< LOQ	<LOD	<LOD	<LOD	<LOD	<LOD	<LOD	<LOD	<LOD	<LOD
Roasted peanut snacks	20230.0S281	132	35.3	0.6	<LOD	<LOD	<LOD	<LOD	<LOD	<LOD	<LOD	<LOD
Roasted peanut snacks	20230.0S282	0.72	< LOQ	<LOD	<LOD	<LOD	<LOD	<LOD	<LOD	<LOD	<LOD	<LOD
Roasted peanut snacks	20230.0S283	<LOD	<LOD	<LOD	<LOD	<LOD	<LOD	<LOD	<LOD	<LOD	<LOD	<LOD
Roasted peanut snacks	20230.0S284	6.1	1	1.7	<LOD	<LOD	<LOD	<LOD	<LOD	<LOD	<LOD	<LOD
Kluiklui	20230.0S307	119.2	25.3	5.8	1.6	<LOD	<LOD	<LOD	<LOD	31.3	22.3	<LOD
Kluiklui	20230.0S308	205.8	42.7	15.9	1.9	<LOD	120.6	28.4	<LOD	20.8	13.9	<LOD
Kluiklui	20230.0S309	254.0	56.9	13.9	1.2	<LOD	<LOD	<LOD	<LOD	12.0	7.0	<LOD
Kluiklui	20230.0S310	498.4	146.5	23.7	2.1	<LOD	< LOQ	< LOQ	<LOD	8.2	14.5	<LOD
Kluiklui	20230.0S316	617.7	105.9	25.5	2.2	<LOD	<LOD	<LOD	<LOD	2.0	0.4	<LOD
Kluiklui	20230.0S317	108.9	24.6	5.0	1.5	<LOD	<LOD	<LOD	<LOD	26.7	16.3	<LOD
Kluiklui	20230.0S318	478.0	<LOD	<LOD	<LOD	<LOD	<LOD	<LOD	<LOD	668.0	909.0	4.0
Kluiklui	20230.0S319	150.7	40.2	13.9	3.3	<LOD	<LOD	<LOD	<LOD	80.6	68.5	<LOD
Kluiklui	20230.0S320	2144.6	416.4	76.8	6.3	<LOD	<LOD	<LOD	<LOD	23.6	13.6	<LOD
Kluiklui	20230.0S322	878.9	204.5	37.1	4.8	<LOD	<LOD	<LOD	<LOD	55.7	32.9	<LOD
Kluiklui	20230.0S327	446.1	86.4	5.9	1.1	<LOD	<LOD	<LOD	<LOD	23.8	24.0	<LOD
Kluiklui	20230.0S328	1395.2	308.7	5.9	1.7	<LOD	<LOD	<LOD	<LOD	31.9	16.3	<LOD
Kluiklui	20230.0S329	320.0	83.9	2.2	< LOQ	<LOD	<LOD	<LOD	<LOD	39.0	29.9	< LOQ

Kluiklui	20230.0S330	740.8	151.2	12.8	3.1	<LOD	<LOD	<LOD	<LOD	76.7	100.8	<LOD
Kluiklui	20230.0S331	760.8	176.0	31.7	4.9	<LOD	<LOD	<LOD	<LOD	16.4	16.0	< LOQ
Kluiklui	20230.0S332	1050.4	233.2	13.0	3.0	<LOD	<LOD	<LOD	<LOD	23.3	17.5	< LOQ
Kluiklui	20230.0S333	723.5	154.7	0.6	<LOD	<LOD	<LOD	<LOD	<LOD	18.3	9.0	<LOD
Kluiklui	20230.0S334	196.0	<LOD	<LOD	<LOD	<LOD	<LOD	<LOD	<LOD	81.0	139.0	4.0
Kluiklui	20230.0S341	92.2	18.9	15.6	2.9	<LOD	<LOD	<LOD	<LOD	1.9	< LOQ	<LOD
Kluiklui	20230.0S342	758.5	186.3	49.9	10.8	<LOD	<LOD	<LOD	<LOD	38.4	18.4	< LOQ
Kluiklui	20230.0S343	394.8	110.6	50.4	13.0	<LOD	<LOD	<LOD	<LOD	2.6	1.8	<LOD
Kluiklui	20230.0S344	144.9	34.6	1.6	<LOD	<LOD	< LOQ	<LOD	<LOD	<LOD	<LOD	<LOD
Kluiklui	20230.0S345	89.1	22.8	<LOD	<LOD	<LOD	<LOD	<LOD	<LOD	<LOD	<LOD	<LOD
Kluiklui	20230.0S347	293.2	61.6	1.9	0.8	<LOD	<LOD	<LOD	<LOD	10.1	8.6	<LOD
Kluiklui	20230.0S348	204.0	38.6	2.3	<LOD	<LOD	<LOD	<LOD	<LOD	<LOD	< LOQ	<LOD
LOQ		*0.2*	*0.2*	*0.2*	*0.2*	*10*	*10*	*10*	*10*	*0.5*	*0.5*	*10*
LOD		*0.02*	*0.07*	*0.16*	*0.13*	*8*	*2.4*	*8*	*6.8*	*0.4*	*0.4*	-

Table S2: Statistics of main mycotoxins concentrations of marketed peanut-based foods (μg/kg, UB)

Sample	Parameter	AFB_1*	AFB_2***	AFG_1***	AFG_2***	AF_{tot}***	OTA***	OTB***
Roasted peanut (n=27)	Sample > LOQ**	18.00	9.00	6.00	1.00	-	3.00	1.00
	Mean ± SD	14.23±39.91[a]	2.05±6.78[a]	0.31±0.36[a]	0.14±0.07[a]	16.74±44.26[a]	189.00±978.48[a]	79.78±412.34[a]
	Min	0.02	0.07	0.16	0.13	0.38	0.40	0.40
	Med	1.00	0.07	0.16	0.13	1.36	0.40	0.40
	P95	97.80	4.83	0.97	0.13	124.71	4.40	0.50
	Max	169.00	35.30	1.70	0.50	169.36	5085.00	2143.00
	% Sample > EU ML	41.00	N/A***	N/A	N/A	33.00	N/A	N/A
Kluiklui (n=25)	Sample > LOQ**	25.00	23.00	22.00	18.0	-	22.0	21.0
	Mean ± SD	522.63±478.48[b]	109.21±102.84[b]	16.47±19.46[b]	2.68±3.25[b]	650.99±591.98[b]	51.74±130.72[b]	59.25±180.04[b]
	Min	89.05	0.07	0.16	0.13	112.13	0.40	0.38
	Med	394.82	83.89	12.79	1.74	478.36	23.26	15.98
	P95	1326.27	293.55	50.28	9.88	1629.10	80.92	131.35
	Max	2144.64	416.44	76.76	13.00	2644.14	668.00	909.00
	% Sample > EU ML	100.00	N/A	N/A	N/A	100.0	N/A	N/A

UB (Upper bound values): concentrations below the limit of quantification (LOQ) and the limit of detection (LOD) were replaced by the LOQ and LOD values.

**AFB1: Aflatoxin B1, AFB2: Aflatoxin B2, AFG1: Aflatoxin G1, AFG2: Aflatoxin G2, AFtot: Total aflatoxin (AFB1+ AFB2+ AFG1+ AFG2), OTA: Ochratoxin A, OTB: Ochratoxin B;*

***Sample > LOQ: Number of samples above limit of quantification (AFB1, AFB2, AFG1, AFG2: 0.2 μg/kg; FUM B1, FUM B2: 10 μg/kg; OTA, OTB: 0.5 μg/kg);*

Maximum limits of 2.0 and 4.0 μg/kg for AFB1 and Total Aflatoxin for peanut-based foods [1];

****No maximum limits (ML) existed for this mycotoxin for peanut-based foods. N/A, not applicable.*

Table S3: Mycotoxins concentration in raw peanut (µg/kg)

Trial number	Municipality	Product	Processor	Sample	AFB1	AFB2	AFG1	AFG2	AOH	FUM B1	FUM B2	OTA	OTB
1	Covè	*Kluiklui*	KP-1	Pool 1	<LOD	<LOD	<LOD	<LOD	<LOD	<LOD	<LOD	<LOD	<LOD
			KP-2										
			KP-3										
		Roasted peanut snack	RP-1	Pool 2	<LOD	<LOD	<LOD	<LOD	<LOD	<LOD	<LOD	<LOD	<LOD
			RP-2										
			RP-3										
	Aplahoué	*Kluiklui*	KP-4	Pool 3	<LOD	<LOD	<LOD	<LOD	<LOD	<LOD	<LOD	<LOD	<LOD
			KP-5										
			KP-6										
	Abomey	Roasted peanut snack	RP-4	Pool 4	<LOD	<LOD	<LOD	<LOD	<LOD	<LOD	<LOD	<LOD	<LOD
			RP-5										
			RP-6										
2	Covè	*Kluiklui*	KP-1	Pool 5	<LOD	<LOD	<LOD	<LOD	<LOD	<LOD	<LOD	<LOD	<LOD
			KP-2										
			KP-3										
		Roasted peanut snack	RP-1	Pool 6	<LOD	<LOD	<LOD	<LOD	<LOD	<LOD	<LOD	<LOD	<LOD
			RP-2										
			RP-3										
	Aplahoué	*Kluiklui*	KP-4	Pool 7	<LOD	<LOD	<LOD	<LOD	<LOD	<LOD	<LOD	<LOD	<LOD
			KP-5										
			KP-6										
	Abomey	Roasted peanut snack	RP-4	Pool 8	<LOD	<LOD	<LOD	<LOD	<LOD	<LOD	<LOD	<LOD	<LOD
			RP-5										
			RP-6										
3	Covè	*Kluiklu*i	KP-1	Pool 9	<LOD	<LOD	<LOD	<LOD	<LOD	<LOD	<LOD	<LOD	<LOD

			KP-2											
			KP-3											
		Roasted peanut snack	RP-1	Pool 10		<LOQ	<LOD	<LOD	<LOD	<LOD	<LOD	<LOD	<LOD	<LOD
			RP-2											
			RP-3											
	Aplahoué	*Kluiklui*	KP-4	Pool 11	2024-S428	<LOD	<LOD	<LOD	<LOD	<LOD	<LOD	<LOD	<LOD	<LOD
			KP-5		2024-S429	1.54	< LOQ	<LOD	<LOD	<LOD	<LOD	<LOD	<LOD	<LOD
			KP-6		2024-S430	0.85	< LOQ	<LOD	<LOD	<LOD	<LOD	<LOD	<LOD	<LOD
	Abomey	Roasted peanut snack	RP-4	Pool 12	2024-S431	< LOQ	<LOD	<LOD	<LOD	<LOD	<LOD	<LOD	< LOQ	<LOD
			RP-5		2024-S432	< LOQ	<LOD	<LOD	<LOD	<LOD	<LOD	<LOD	< LOQ	<LOD
			RP-6		2024-S433	2.63	0.34	<LOD	<LOD	<LOD	<LOD	<LOD	<LOD	<LOD

Table S4: Mycotoxins contamination (µg/kg) in samples collected during *kluiklui* production

Trial number	Process	Processor	Sample ID	Sample type	AFB1	AFB2	AFG1	AFG2	AOH	FUM B1	FUM B2	OTA	OTB
1	Without maize flour (Covè municipality)	KP-1, 2, 3	Pool 1	Raw peanut	<LOD	<LOD	<LOD	<LOD	<LOD	<LOD	<LOD	<LOD	<LOD
		KP-1	2024-S452	Peanut paste	48.77	6.91	4.5	< LOQ	28.79	<LOD	<LOD	3.03	2.41
			2024-S488	Kluiklui	53.9	7.22	4.35	1.06	27.76	<LOD	<LOD	7.67	5.03
		KP-2	2024-S453	Peanut paste	15.65	3.64	1.2	<LOD	<LOD	<LOD	<LOQ	5.16	1.6
			2024-S489	Kluiklui	37.8	7.16	<LOQ	<LOQ	<LOQ	<LOQ	<LOQ	4.3	1.22
		KP-3	2024-S454	Peanut paste	25.31	3.65	<LOD	<LOD	<LOD	<LOD	<LOD	1.75	0.55
			2024-S490	Kluiklui	29.57	7.65	1.69	<LOD	<LOD	<LOD	<LOD	7.8	2.33
	With maize flour (Aplahoué municipality)	KP-4, 5, 6	Pool 3	Raw peanut	<LOD	<LOD	<LOD	<LOD	<LOD	<LOD	<LOD	<LOD	<LOD
		KP-4	2024-S456	Peanut paste	36.22	6.37	0.96	<LOD	<LOD	<LOD	<LOD	<LOD	< LOQ
			2024-S491	Kluiklui	35	6.6	0.9	<LOD	<LOD	<LOD	<LOD	<LOD	<LOD
		KP-5	2024-S455	Peanut paste	2.44	0.4	<LOD	<LOD	<LOD	<LOD	<LOD	4.88	4.2
			2024-S492	Kluiklui	2.22	0.51	<LOD	<LOD	<LOD	<LOD	<LOD	7.41	0.78
		KP-6	2024-S457	Peanut paste	4.61	0.71	<LOD	<LOD	<LOD	<LOD	<LOD	8.13	< LOQ
			2024-S493	Kluiklui	2.02	0.53	<LOD	<LOD	<LOD	104.4	34.06	8.22	0.72
2	Without maize flour (Covè municipality)	KP-1, 2, 3	Pool 5	Raw peanut	<LOD	<LOD	<LOD	<LOD	<LOD	<LOD	<LOD	<LOD	<LOD
		KP-1	2024-S458	Peanut paste	111.73	17.74	3.04	< LOQ	<LOD	<LOD	<LOD	6.64	2.19
			2024-S494	Kluiklui	2.52	0.47	<LOD	<LOD	<LOD	< LOQ	< LOQ	10.32	2.23

		KP-2	2024-S459	Peanut paste	36.99	6.56	7.54	1.78	<LOD	<LOD	<LOD	5.82	3.19
			2024-S495	Kluiklui	25.36	4.96	4.55	<LOD	<LOD	<LOD	<LOD	5.57	3.1
		KP-3	2024-S460	Peanut paste	41.85	6.3	0.71	<LOD	<LOD	<LOD	<LOD	8.6	4.85
			2024-S496	Kluiklui	18.47	3.71	<LOD	<LOD	<LOD	<LOD	<LOD	24.33	13.68
	With maize flour (Aplahoué municipality)	KP-4, 5, 6	Pool 7	Raw peanut	<LOD	<LOD	<LOD	<LOD	<LOD	<LOD	<LOD	<LOD	<LOD
		KP-4	2024-S524	Maize flour	11.25	1.29	1.51	<LOD	<LOD	492.68	164.7	< LOQ	<LOD
			2024-S461	Peanut paste	8.42	0.83	<LOD	<LOD	<LOD	<LOD	<LOD	<LOD	<LOD
			2024-S497	Kluiklui	3.15	0.52	<LOD	<LOD	<LOD	< LOQ	< LOQ	11.15	2.68
		KP-5	2024-S525	Maize flour	2.79	0.5	0.23	<LOD	<LOD	685.97	232.41	3.5	1.02
			2024-S462	Peanut paste	4.53	1.31	<LOD	<LOD	<LOD	<LOD	<LOD	19.79	18.01
			2024-S498	Kluiklui	3.27	1.29	<LOD	<LOD	<LOD	<LOD	<LOD	20.41	33.43
		KP-6	2024-S526	Maize flour	0.65	0.15	<LOD	<LOD	<LOD	302.19	100.56	<LOD	<LOD
			2024-S463	Peanut paste	3.81	0.56	<LOD	<LOD	<LOD	<LOD	<LOD	20.35	1.65
			2024-S499	Kluiklui	2.34	0.53	<LOD	<LOD	<LOD	39.32	< LOQ	11.55	1.37
3	Without maize flour (Covè municipality)	KP-1, 2, 3	Pool 9	Raw peanut	<LOD	<LOD	<LOD	<LOD	<LOD	<LOD	<LOD	<LOD	<LOD
		KP-1	2024-S464	Peanut paste	57.84	6.65	<LOD	<LOD	<LOD	<LOD	<LOD	1.46	<LOQ
			2024-S500	Kluiklui	106.53	21.88	0.83	<LOD	<LOD	< LOQ	<LOD	2.69	1.94
		KP-2	2024-S465	Peanut paste	52.44	8.2	<LOD	<LOD	<LOD	<LOD	<LOD	<LOD	<LOD
			2024-S501	Kluiklui	22.33	4.17	<LOD	<LOD	<LOD	<LOD	<LOD	<LOD	<LOD
		KP-3	2024-S466	Peanut paste	64.47	10.73	2.36	<LOD	<LOD	<LOD	<LOD	<LOD	<LOD

			2024-S502	Kluiklui	43.57	9.13	2.32	<LOD	<LOD	<LOD	<LOD	<LOD	<LOD
	With maize flour (Aplahoué municipality)	KP-4	2024-S428	Raw peanut	<LOD	<LOD	<LOD	<LOD	<LOD	<LOD	<LOD	<LOD	<LOD
			2024-S527	Maize flour	63.78	6.21	<LOD	<LOD	<LOD	1822.42	601.59	< LOQ	<LOD
			2024-S467	Peanut paste	32.34	5.01	<LOD	<LOD	<LOD	<LOD	<LOD	<LOD	<LOD
			2024-S503	Kluiklui	21.59	6.15	<LOD	<LOD	<LOD	<LOD	< LOQ	<LOD	<LOD
		KP-5	2024-S429	Raw peanut	1.54	<LOQ	<LOD	<LOD	<LOD	<LOD	<LOD	<LOD	<LOD
			2024-S528	Maize flour	7.88	0.79	<LOD	<LOD	<LOD	738.9	274.63	8.59	1.08
			2024-S468	Peanut paste	21.35	41.01	<LOD	<LOD	<LOD	<LOD	<LOD	<LOD	<LOD
			2024-S504	Kluiklui	18.03	16.99	<LOD	<LOD	<LOD	<LOD	<LOD	<LOD	<LOD
		KP-6	2024-S430	Raw peanut	0.85	<LOQ	<LOD	<LOD	<LOD	<LOD	<LOD	<LOD	<LOD
			2024-S529	Maize flour	58.32	6.7	0.27	<LOD	<LOD	1906.9	629.12	<LOD	<LOD
			2024-S469	Peanut paste	18.11	2.99	<LOD	<LOD	<LOD	<LOD	<LOD	<LOD	<LOD
			2024-S505	Kluiklui	17.97	4.1	<LOD	<LOD	<LOD	115.79	31.2	<LOD	<LOD
				LOQ	*0.2*	*0.2*	*0.2*	*0.2*	*10*	*10*	*10*	*0.5*	*0.5*
				LOD	*0.02*	*0.07*	*0.16*	*0.13*	*8*	*2.4*	*8*	*0.4*	*0.4*

Table S5: Mycotoxins concentration in roasted peanut snacks (µg/kg)

Trial number	Process	Processor	Sample ID	Sample type	AFB1	AFB2	AFG1	AFG2	AOH	FUM B1	FUM B2	OTA	OTB
1	Roasting without heat transfer material (Covè municipality)	RP-1,2,3	Pool 2	Raw peanut	<LOD	<LOD	<LOD	<LOD	<LOD	<LOD	<LOD	<LOD	<LOD
		RP-1	2024-S509	Roasted peanut snack	0.2	< LOQ	<LOD	<LOD	<LOD	<LOD	<LOD	<LOD	<LOD
		RP-2	2024-S510	Roasted peanut snack	<LOD	<LOD	<LOD	<LOD	<LOD	<LOD	<LOD	<LOD	<LOD
		RP-3	2024-S511	Roasted peanut snack	< LOQ	<LOD	<LOD	<LOD	<LOD	<LOD	<LOD	<LOD	<LOD
	Roasting without heat transfer material (Covè municipality)	RP-4,5,6	Pool 4	Raw peanut	<LOD	<LOD	<LOD	<LOD	<LOD	<LOD	<LOD	<LOD	<LOD
		RP-4	2024-S506	Roasted peanut snack	<LOD	<LOD	<LOD	<LOD	<LOD	<LOD	<LOD	<LOD	<LOD
		RP-5	2024-S507	Roasted peanut snack	<LOD	<LOD	<LOD	<LOD	<LOD	<LOD	<LOD	<LOD	<LOD
		RP-6	2024-S508	Roasted peanut snack	<LOD	<LOD	<LOD	<LOD	<LOD	<LOD	<LOD	<LOD	<LOD
2	Roasting without heat transfer material (Covè municipality)	RP-1,2,3	Pool 6	Raw peanut	<LOD	<LOD	<LOD	<LOD	<LOD	<LOD	<LOD	<LOD	<LOD
		RP-1	2024-S512	Roasted peanut snack	0.2	< LOQ	<LOD	<LOD	<LOD	<LOD	<LOD	<LOD	< LOQ
		RP-2	2024-S513	Roasted peanut snack	< LOQ	< LOQ	<LOD	<LOD	<LOD	<LOD	<LOD	<LOD	<LOD
		RP-3	2024-S514	Roasted peanut snack	<LOD	<LOD	<LOD	<LOD	<LOD	<LOD	<LOD	<LOD	< LOQ
	Roasting with white clay as heat transfer	RP-4,5,6	Pool 8	Raw peanut	<LOD	<LOD	<LOD	<LOD	<LOD	<LOD	<LOD	<LOD	<LOD
		RP-4	2024-S515	Roasted peanut snack	<LOD	<LOD	<LOD	<LOD	<LOD	<LOD	<LOD	<LOD	< LOQ

	material (Abomey municipality)	RP-5	2024-S516	Roasted peanut snack	<LOD	<LOD	<LOD	<LOD	<LOD	<LOD	<LOD	<LOD	< LOQ
		RP-6	2024-S517	Roasted peanut snack	<LOD	<LOD	<LOD	<LOD	<LOD	<LOD	<LOD	<LOD	< LOQ
3	Roasting without heat transfer material (Covè municipality)	RP-1	2024-S425	Raw peanut	<LOD	<LOD	<LOD	<LOD	<LOD	<LOD	<LOD	<LOD	<LOD
			2024-S518	Roasted peanut snack	0.49	0.22	<LOD	<LOD	<LOD	<LOD	<LOD	<LOD	<LOD
		RP-2	2024-S426	Raw peanut	< LOQ	<LOD	<LOD	<LOD	<LOD	<LOD	<LOD	<LOD	<LOD
			2024-S519	Roasted peanut snack	2.27	0.59	<LOD	<LOD	<LOD	<LOD	<LOD	<LOD	<LOD
		RP-3	2024-S427	Raw peanut	< LOQ	<LOD	<LOD	<LOD	<LOD	<LOD	<LOD	<LOD	<LOD
			2024-S520	Roasted peanut snack	0.92	< LOQ	<LOD	<LOD	<LOD	<LOD	<LOD	<LOD	<LOD
	Roasting with white clay as heat transfer material (Abomey municipality)	RP-4	2024-S431	Raw peanut	< LOQ	<LOD	<LOD	<LOD	<LOD	<LOD	<LOD	< LOQ	<LOD
			2024-S521	Roasted peanut snack	<LOD	<LOD	<LOD	<LOD	<LOD	<LOD	<LOD	<LOD	<LOD
		RP-5	2024-S432	Raw peanut	< LOQ	<LOD	<LOD	<LOD	<LOD	<LOD	<LOD	< LOQ	<LOD
			2024-S522	Roasted peanut snack	0.7	0.2	<LOD	<LOD	<LOD	<LOD	<LOD	<LOD	<LOD
		RP-6	2024-S433	Raw peanut	2.63	0.34	<LOD	<LOD	<LOD	<LOD	<LOD	<LOD	<LOD
			2024-S523	Roasted peanut snack	<LOQ	0.3	<LOD	<LOD	<LOD	<LOD	<LOD	<LOD	<LOD
				LOQ	*0.2*	*0.2*	*0.2*	*0.2*	*10*	*10*	*10*	*0.5*	*0.5*
				LOD	*0.02*	*0.07*	*0.16*	*0.13*	*8*	*2.4*	*8*	*0.4*	*0.4*

Table S6: Mean mycotoxin concentrations in marketed and just-produced peanut-based foods (μg/kg UB)

**Samples**		**AFB1**	**AF tot**	**OTA**
Kluiklui	Marketed (n=27)	522.63±478.48[a]	651.03±591.97[a]	52.89±130.28[a]
	Just produced (n=18)	24.76±25.77[b]	31.67±31.27[b]	6.91±6.92[b]
Roasted peanut snacks	Marketed (n=25)	14.26±39.89[1]	16.94±39.89[1]	189.07±978.46[1]
	Just produced (n=6)	0.80±0.77[1]	1.33±0.77[1]	0.50±0.00[1]

Values within the same column followed by the same letter or number do not differ significantly at the 5% probability level. The mean concentrations of AFB1 (p= 2.158e-06), total aflatoxins (p=1.924e-06) and OTA (p=0.001096) were significantly higher in marketed kluiklui. The mean concentrations of AFB1 (p= 0.3789), *total aflatoxins (p=0.2184) and OTA* (p=0.4264) *were not significantly different in marketed and just-produced roasted peanut.*

Table S7: Detection of aflatoxigenic and non-aflatoxigenic *A. flavus* isolates in samples collected during *kluiklui* production

Sample	Number of *Aspergillus flavus*	Number of positive strains	Number of negatives strains
Raw peanuts (n=18)	1	1	-
Peanut paste (n=18)	34	31	3
Maize flour (n=6)	1	1	0
Kluiklui (n=18)	10	9	1

Table S8: Pooling of raw material (peanut seeds) used for *kluiklu*i and roasted peanut snack processing

<table>
<tr><th>Trial number</th><th>Municipality</th><th>Product</th><th>Processor</th><th>Sample ID</th><th>Pools</th></tr>
<tr><td rowspan="12">1</td><td rowspan="6">Covè</td><td rowspan="3">Kluiklui</td><td>KP-1</td><td>2024-S398</td><td rowspan="3">Pool 1</td></tr>
<tr><td>KP-2</td><td>2024-S399</td></tr>
<tr><td>KP-3</td><td>2024-S400</td></tr>
<tr><td rowspan="3">Roasted peanut snack</td><td>RP-1</td><td>2024-S401</td><td rowspan="3">Pool2</td></tr>
<tr><td>RP-2</td><td>2024-S402</td></tr>
<tr><td>RP-3</td><td>2024-S403</td></tr>
<tr><td rowspan="3">Aplahoué</td><td rowspan="3">Kluiklui</td><td>KP-4</td><td>2024-S404</td><td rowspan="3">Pool 3</td></tr>
<tr><td>KP-5</td><td>2024-S405</td></tr>
<tr><td>KP-6</td><td>2024-S406</td></tr>
<tr><td rowspan="3">Abomey</td><td rowspan="3">Roasted peanut snack</td><td>RP-4</td><td>2024-S407</td><td rowspan="3">Pool4</td></tr>
<tr><td>RP-5</td><td>2024-S408</td></tr>
<tr><td>RP-6</td><td>2024-S409</td></tr>
<tr><td rowspan="12">2</td><td rowspan="6">Covè</td><td rowspan="3">Kluiklui</td><td>KP-1</td><td>2024-S410</td><td rowspan="3">Pool 5</td></tr>
<tr><td>KP-2</td><td>2024-S411</td></tr>
<tr><td>KP-3</td><td>2024-S412</td></tr>
<tr><td rowspan="3">Roasted peanut snack</td><td>RP-1</td><td>2024-S413</td><td rowspan="3">Pool 6</td></tr>
<tr><td>RP-2</td><td>2024-S414</td></tr>
<tr><td>RP-3</td><td>2024-S415</td></tr>
<tr><td rowspan="3">Aplahoué</td><td rowspan="3">Kluiklui</td><td>KP-4</td><td>2024-S416</td><td rowspan="3">Pool 7</td></tr>
<tr><td>KP-5</td><td>2024-S417</td></tr>
<tr><td>KP-6</td><td>2024-S418</td></tr>
<tr><td rowspan="3">Abomey</td><td rowspan="3">Roasted peanut snack</td><td>RP-4</td><td>2024-S419</td><td rowspan="3">Pool 8</td></tr>
<tr><td>RP-5</td><td>2024-S420</td></tr>
<tr><td>RP-6</td><td>2024-S421</td></tr>
<tr><td rowspan="12">3</td><td rowspan="6">Covè</td><td rowspan="3">Kluiklui</td><td>KP-1</td><td>2024-S422</td><td rowspan="3">Pool 9</td></tr>
<tr><td>KP-2</td><td>2024-S423</td></tr>
<tr><td>KP-3</td><td>2024-S424</td></tr>
<tr><td rowspan="3">Roasted peanut snack</td><td>RP-1</td><td>2024-S425</td><td rowspan="3">Pool 10</td></tr>
<tr><td>RP-2</td><td>2024-S426</td></tr>
<tr><td>RP-3</td><td>2024-S427</td></tr>
<tr><td rowspan="3">Aplahoué</td><td rowspan="3">Kluiklui</td><td>KP-4</td><td>2024-S428</td><td rowspan="3">Pool 11</td></tr>
<tr><td>KP-5</td><td>2024-S429</td></tr>
<tr><td>KP-6</td><td>2024-S430</td></tr>
<tr><td rowspan="3">Abomey</td><td rowspan="3">Roasted peanut snack</td><td>RP-4</td><td>2024-S431</td><td rowspan="3">Pool 12</td></tr>
<tr><td>RP-5</td><td>2024-S432</td></tr>
<tr><td>RP-6</td><td>2024-S433</td></tr>
</table>

Reference

1. European Commission. Commission Regulation (EU) 2023/915 of 25 April 2023 on maximum levels for certain contaminants in food and repealing Regulation (EC) No 1881/2006 (Text with EEA relevance). **2023**, *C/2023/35*, 103–157.
